# Complete resection of rectal cancer with a synchronous solitary adrenal metastasis: A case report

**DOI:** 10.1016/j.ijscr.2020.09.094

**Published:** 2020-09-16

**Authors:** Hirokatsu Hayashi, Yusuke Murase, Hitoya Sano, Kimitosi Nishio, Iwao Kumazawa

**Affiliations:** Department of Surgery, JA GIFU Kouseiren Ibi Kosei Hospital, 2547-4 Miwa, Ibigawa-cho, Ibi District, Gifu Prefecture, 501-0696, Japan

**Keywords:** CT, computed tomography, PET-CT, positron emission tomography–computed tomography, PR, partial response, Colorectal cancer, Adrenal metastasis, Complete resection

## Abstract

**Introduction:**

Solitary adrenal metastasis derived from colorectal cancer is rare. Adrenal metastasis is usually associated with systemic spread of the disease and is considered to be unsuitable for surgical resection. However, it has been reported that an aggressive surgical resection of adrenal metastasis results in improved overall survival in selected patients. We herein report an extremely rare case of complete resection of rectal cancer with a synchronous solitary adrenal metastasis.

**Presentation of case:**

A 70-year-old woman who presented with bloody stool was diagnosed with rectal cancer with a synchronous solitary adrenal metastasis and was suspected of having liver invasion. After a total of 2 cycles of chemotherapy with capecitabine and oxaliplatin, an abdominoperineal resection with D2 (proxD3) lymph node dissection and right adrenalectomy was performed and complete resection was possible. According to the TNM classification, the diagnosis was stage IVA (fT3N1bM1a[ADR]). At 18 months after surgery, the patient is alive with no evidence of recurrence and distant metastasis.

**Discussion:**

Complete resection of adrenal metastasis may have a possibility of leading to a good prognosis in patients with a synchronous solitary adrenal metastasis.

**Conclusion:**

Adrenalectomy should be considered in patients who can undergo complete resection, as it may offer a good long-term prognosis.

## Highlights

• Adrenal metastasis is usually associated with systemic spread of the disease and is considered to be unsuitable for surgical resection.
• Adrenalectomy should be considered in patients who can undergo complete resection.
• Adrenalectomy may offer a better long-term prognosis.

## 1 Introduction

Solitary adrenal metastasis derived from colorectal cancer is rare. Adrenal metastasis usually associated with systemic spread of the disease and is considered to be unsuitable for surgical resection. However, it has been reported that an aggressive surgical resection of adrenal metastasis results in improved overall survival in selected patients [1]. We herein present an extremely rare case of synchronous solitary adrenal metastasis derived from rectal cancer. This work was reported in line with the Surgical Case Report criteria [2].

## 2 Presentation of case

**Fig. 1:**
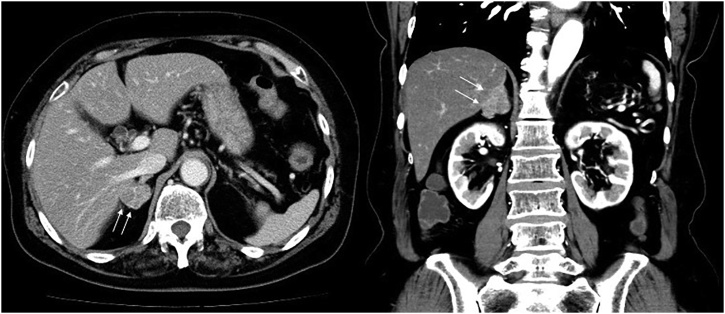
Contrast-enhanced computed tomography findings at the time of diagnosis. The images show an enlargement of the right adrenal gland that was 26 mm in size (arrows).

**Fig. 2:**
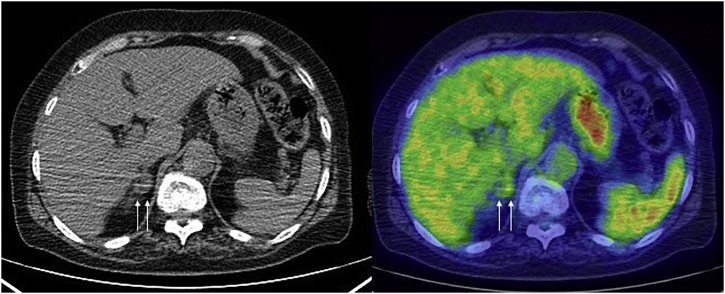
Positron emission tomography–computed tomography scan after chemotherapy. The images show that the right adrenal metastasis decreased in size to 13 mm (arrows).

A 70-year-old woman presented with bloody stool. She had a 2-month history of blood in her feces but without abdominal pain or any relevant past or family history. A colonoscopy revealed a type 2 tumor in the rectum and subsequent biopsy specimens were obtained. Histopathological examination showed a moderately differentiated adenocarcinoma of rectal origin with RAS mutation. Her serum carcinoembryonic antigen and carbohydrate antigen 19-9 levels were 13.6 and 129.6 ng/mL, respectively. Other laboratory data showed no abnormalities. A computed tomography (CT) scan revealed circumferential thickness of the rectum and an enlargement of the right adrenal gland with a size of 26 mm; the patient was suspected of having liver invasion (Fig. 1). There was no evidence of metastasis in other organs. The diagnosis was rectal cancer with a synchronous solitary adrenal metastasis. Chemotherapy with capecitabine and oxaliplatin was administered prior to surgery. However, chemotherapy was discontinued in the third cycle because of anaphylactic shock caused by oxaliplatin. After a total of 2 cycles of chemotherapy, the right adrenal metastasis had decreased in size to 13 mm (Response Evaluation Criteria in Solid Tumors: partial response), and new distant metastases were not detected on a positron emission tomography–CT scan (Fig. 2). At 3 months after diagnosis, an abdominoperineal resection with D2 (proxD3) lymph node dissection and a right adrenalectomy was performed. Based on the surgical findings, rectal cancer with regional lymph nodes and adrenal metastasis without liver invasion could be resected completely; no other distant metastases were observed. The pathological examination revealed a moderately differentiated type of tubular adenocarcinoma in rectal cancer penetrating the muscularis propria with lymphatic and vascular invasion and metastatic involvement in 2 of 9 dissected lymph nodes; the adenocarcinoma was similar to the rectal cancer found in the adrenal gland, which was consistent with the adrenal metastasis derived from rectal cancer. There was no evidence of liver invasion. As a histological response to chemotherapy, a quarter of the area around the viable tumor has been replaced by fibers in the rectal cancer (Grade 1a). According to the TNM Classification of Malignant Tumors, the diagnosis was stage IVA (fT3N1bM1a[ADR]). Chemotherapy with capecitabine was administered every 3 weeks for 12 months after surgery. At 18 months after surgery, the patient is alive with no evidence of recurrence and distant metastasis.

## 3 Discussion

Although there is no clear evidence of survival benefit for surgical resection of adrenal metastasis derived from colorectal cancer, it has been reported that adrenalectomy may improve long-term outcomes. Vazquez et al. reported that an aggressive surgical approach results in improved overall survival in patients with adrenal metastasis derived from soft tissues, kidney, lung, and pancreas. Other tumors may benefit from such approach [1]. Muth et al. reported that, in 30 patients who underwent adrenalectomy for metastasis between 1996 and 2007, including five cases of colorectal cancer, the independent prognosticators of favorable survival included adrenalectomy as a potential cure, no previous metastasis surgeries, and tumor type (colorectal cancer and renal cell carcinoma) [3]. In a study by Kim et al. involving 37 patients who underwent adrenalectomy for metastasis between 1986 and 1996, including five cases of colorectal cancer, the predictors of improved survival were disease-free intervals >6 months and complete resections [4]. Shimomura et al. showed the survival benefit of metastasectomy in patients with stage IV colorectal cancer, and they reported that the 5-year overall survival rates of R0, R1, and R2 were 45.9%, 12.5%, and 6.7%, respectively [5]. In the present case, the patient had a solitary adrenal metastasis and no other distant metastases; thus, it was determined that a complete resection may be possible. However, radiological imaging revealed that the adrenal metastasis may have contacted and invaded the liver. Therefore, we decided to perform chemotherapy prior to surgery to avoid resection of the liver and ensure adequate surgical margins. As a result, no liver invasion was observed and a complete resection was possible.

A similar case report in Japan found that, out of 53 cases of adrenal metastasis derived from colorectal cancer, 18 cases were solitary adrenal metastases, and the other cases were part of systemic metastases [[6], [7], [8], [9], [10], [11], [12], [13], [14], [15]]. Fourteen cases had disease-free intervals >6 months, which means that they were metachronous [[6], [7], [8], [9], [10], [11]]. Five cases had disease-free intervals <6 months, which means that they were synchronous (Table 1, Table 2) [[12], [13], [14], [15]]. In patients with metachronous solitary adrenal metastasis, disease-free intervals ranged from 6 to 38 months, with a median of 15 months. Three cases (21%) had recurrences within the first year after adrenalectomy. Patient survival over 4 years after adrenalectomy was achieved in six cases (42%). Long-term survival can be expected for patients with metachronous solitary adrenal metastasis, whereas only one case (20%) of synchronous solitary adrenal metastasis had survived after adrenalectomy over a four-year period. Moreover, two cases (40%) had recurrence within the first year after adrenalectomy. These results suggest that synchronous solitary adrenal metastasis may have a poorer prognosis than heterochronous solitary adrenal metastasis. However, in both cases, surgery should be considered, as some patients have a good long-term prognosis with adrenalectomy.

**Table 1:** Clinical characteristics of the patients with heterochronous solitary adrenal metastasis.

					Primary			Adrenal metastasis			
No.	Author	Year	Age	Sex	Site	Pathology	Stage	Side	DAR	Prognosis	DMA
1	Fujii [6]	1995	57	M	A	ss,n0,tub1,ly0,v1	II	R	3y2m	9y0m/alive	
2	Nakaguchi [6]	1995	60	F	R	a2,n0,tub2,ly0,v0	II	L	1y9m	4y0m/alive	
3	Mizutani [7]	1995	58	M	R	a2,n0,tub1,ly2,v1	II	R	1y5m	2y7m/dead	4m : lung, prostate gland
4	Itoh [8]	1995	65	F	C	ss,n0,tub2,ly1,v0	II	L	0y6m	0y6m/dead	3m : Inguinal lymph node
5	Ozawa [6]	1996	46	M	D	ss,n1,tub2,ly1,v1	IIIa	R	1y3m	1y2m/alive	
6	Watahiki [6]	1998	61	M	D	ss,n0,tub1 > muc,ly1,v1	II	R	0y7m	4y2m/alive	
7	Emoto [6]	1998	63	F	R	ai,n1,tub2	IIIa	L	1y3m	0y7m/alive	
8	Tokuhara [6]	2002	58	F	A	se,n2,tub2,ly3,v3	IIIb	L	1y0m	0y6m/alive	
9	Kato [6]	2004	67	F	R	se,n0,tub2,ly1,v0	II	R	0y8m	4y0m/alive	
10	Tokuda [6]	2005	56	M	R	ss,n0,tub2,ly2,v1	II	R	1y5m	1y1m/alive	
11	Kurashima [6]	2007	61	F	R	se,n2,tub2,ly1,v1	IIIb	R	1y0m	0y7m/alive	
12	Inaoka [9]	2015	65	F	R	mp,n0,tub1,ly1,v0	I	R	1y7m	12y8m/alive	
13	Yuge [10]	2015	77	F	T	ss,n1,por1 > tub2,ly1,v1	IIIa	L	1y0m	1y0m/alive	
14	Uchiyama [11]	2017	60	M	R	ss,n1,tub1,ly3,v3	IIIa	R	2y1m	10y10m/alive	3m : lung

M:male, F:female, C:cecum, A:ascendingcolon, T:transversecolon, D:descendingcolon, S:sigmoidcolon, R:rectum, y:year, m:month, DAR: duration for adrenal metastasis from resection of colorectal cancer, DMA: duration for metastasis from adrenalectomy.

**Table 2:** Clinical characteristics of the patients with synchronous solitary adrenal metastasis.

					Primary			Adrenal metastasis			
No.	Author	Year	Age	Sex	Site	Pathology	Stage	Side	TA	Prognosis	DMA
1	Kamasako [12]	1995	71	F	S	si,n1,tub1,ly2,v2	IV	R	synchronous	0y11m/alive	8m : liver
2	Ozawa [13]	2002	65	F	R	sei,n2,tub2,ly1,v1	IV	R	synchronous	1y0m/alive	
3	Sugimoto [14]	2017	65	F	R	a,n1,tub1,ly0,v1	IV	R	4m later	1y4m/alive	3m : lung
4	Nakasone [15]	2018	62	F	C	ss,n2,tub2,ly0,v2	IV	L	synchronous	4y0m/alive	
5	Hayashi	2020	70	F	R	ss,n1,tub2,ly1,v1	IV	R	synchronous	1y6m/alive	

M:male, F:female, C:cecum, A:ascendingcolon, T:transversecolon, D:descendingcolon, S:sigmoidcolon, R:rectum, y:year, m:month, TA: time of adrenalectomy, DMA: duration for metastasis from adrenalectomy.

The present report was associated with a few limitations. The observation period was rather short and the number of cases was too small to examine the long-term prognosis. In the future, it is expected that a larger cohort of adrenal metastasis cases will be collected and the long-term prognoses will be clarified.

## 4 Conclusion

Adrenalectomy should be considered in patients who can undergo complete resection, as it may offer a better long-term prognosis.

## Declaration of Competing Interest

The authors declare no conflicts of interest.

## Funding

This research did not receive any specific grant from funding agencies in the public, commercial, or not-for-profit sectors.

## Ethical approval

This report was reviewed and approved by the Institutional Review Board of JA GIFU Kouseiren Ibi Kosei Hospital.

## Consent

Informed consent was obtained from the patient for publication of this case report.

## Author contribution

Hirokatsu Hayashi: Data Acquisition, Data Interpret and writing of the manuscript.

Yusuke Murase & Hitoya Sano e: management of case.

Kimitosi Nishio: Supervision, review and editing.

Iwao Kumazawa: Supervision, review, editing, and final approval of the version to be submitted.

## Registration of research studies

NA.

## Guarantor

The Guarantor is Hirokatsu Hayashi.

## Provenance and peer review

Not commissioned, externally peer-reviewed.
